# Addison’s disease triggered by infection with *mycobacterium abscessus*, but not by adrenal tuberculosis or MAC pulmonary disease, in a subject with type 2 diabetes mellitus: case report

**DOI:** 10.1186/s12902-022-01178-1

**Published:** 2022-10-23

**Authors:** Hideaki Kaneto, Fuminori Tatsumi, Mana Ohnishi, Yuichiro Iwamoto, Yukino Katakura, Masashi Shimoda, Shuhei Nakanishi, Kohei Kaku, Tomoatsu Mune

**Affiliations:** Department of Diabetes, Endocrinology and Metabolism, Kawasaki Medical School, 577 Matsushima, Kurashiki, 701-0192 Japan

**Keywords:** Case report, Addison’s disease, *Mycobacterium abscessus*, Non-tuberculous mycobacterial infection, *Mycobacterium avium* complex pulmonary disease

## Abstract

**Background:**

Addison’s disease is primary adrenal dysfunction and is characterized by decrease of cortisol level and increase of adrenocorticotropic hormone (ACTH) level. It is known that infection is one of main causes of Addison’s disease. Among various infections, tuberculous infection accounts for the majority of them. Recently the number of subjects with non-tuberculous mycobacterial infection has been increased, and the infection can also bring about Addison’s disease. *Mycobacterium avium* complex (MAC) pulmonary disease accounts for the majority of non-tuberculous mycobacterial infection.

**Case presentation:**

An 83-year-old female was suspected of having adrenal failure in our outpatient care and hospitalized in our institution. There was pigmentation in her face, hands and legs, especially in auricle and nail beds in her hands and legs. In rapid ACTH load test (0.25 mg of 1–24 ACTH), cortisol level was not increased at all. An abdominal computed tomography (CT) showed swelling of both adrenal glands accompanied by calcification. QuantiFERON test was negative and *mycobacterium tuberculosis* complex was negative in PCR test using bronchial lung lavage fluid. These data ruled out the possibility of adrenal tuberculosis. It is known that MAC pulmonary disease accounts for the majority of non-tuberculous mycobacterial infection. In this subject, however, anti-MAC antibody was negative and MAC-related bacteria were not detected in PCR test using bronchial lung lavage fluid. These data ruled out the possibility of MAC pulmonary disease. *Mycobacterium abscessus* (Mab) was positive in bronchial lung lavage fluid culture. Based on these data, we diagnosed this subject with Addison’s disease triggered by infection with *mycobacterium abscessus*, but not by adrenal tuberculous or MAC pulmonary disease. Decreased sodium level and increased eosinophil number were normalized and appetite loss was markedly mitigated after starting hydrocortisone therapy. A chest CT which was taken about 6 months later showed drastic reduction of consolidation in the upper lobe of the left lung although calcification in the adrenal gland was still observed.

**Conclusions:**

We should bear in mind the possibility of Addison’s disease triggered by another type of infection rather than adrenal tuberculosis or MAC pulmonary disease.

## Background

Addison’s disease is primary adrenal dysfunction and is characterized by marked decrease of cortisol level accompanied by increase of adrenocorticotropic hormone (ACTH) level. Hyponatremia, hypoglycemia and hypotension are often observed in subjects with Addison’s disease. Pigmentation in face and hands is sometimes observed, although the frequency of pigmentation is relatively low. It is thought that such pigmentation is brought about by the increment of ACTH and/or melanocyte-stimulating hormone through the cortisol deficiency-mediated feedback system.

It is known that infection is one of main causes of Addison’s disease. Among various infections, tuberculous infection accounts for the majority of them [1]. In subjects with typical Addison’s disease due to adrenal gland tuberculous, swelling of both adrenal glands together with calcification is observed in some imaging examination. Recently, the number of subjects with non-tuberculous mycobacterial infection has been increasing [2]. It is known that the infection can also bring about Addison’s disease whose characteristics are similar to those of adrenal gland tuberculosis [3]. In Japan, *mycobacterium avium* complex (MAC) pulmonary disease after infection with *mycobacterium avium* or *mycobacterium intracellulare* accounts for the majority (more than 90%) of non-tuberculous mycobacterial infection [4, 5].

Here we show a subject who had non-tuberculous mycobacterial infection and Addison’s disease triggered by the infection. Furthermore, while MAC pulmonary disease accounts for the majority of non-tuberculous mycobacterial infection, pathogen bacteria in this subject were *mycobacterium abscessus* (Mab), but not MAC. It is noted here that Mab is very rare as a cause of Addison’s disease.

## Case presentation

An 83-year-old female noticed pigmentation in her face, hands and legs. In particular, there was dark pigmentation in auricle and nail beds in her hands and legs. She had appetite loss, general fatigue and a sense of exhaution. Her body weight was reduced by 10 kg for about 1 year. She was suspected of having adrenal failure in our outpatient care and hospitalized in our institution.

**Fig. 1:**
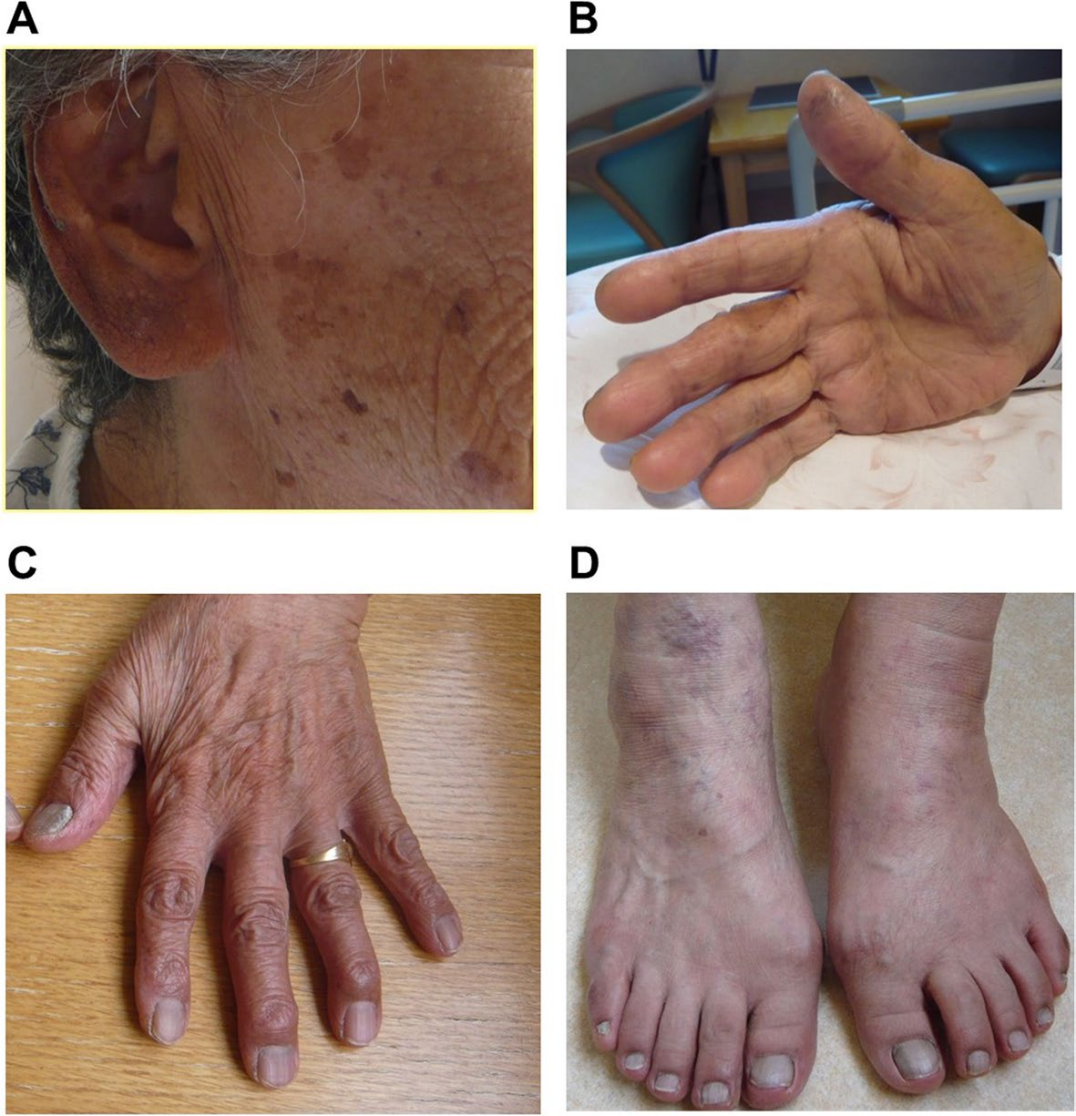
The picture of face (**A**), palm (**B**), and nail beds in her hands and legs (**C**, **D**) in this subject. There was pigmentation in her face, hands and legs. In particular, dark pigmentation was observed in auricle and nail beds in her hands and legs

**Fig. 2:**
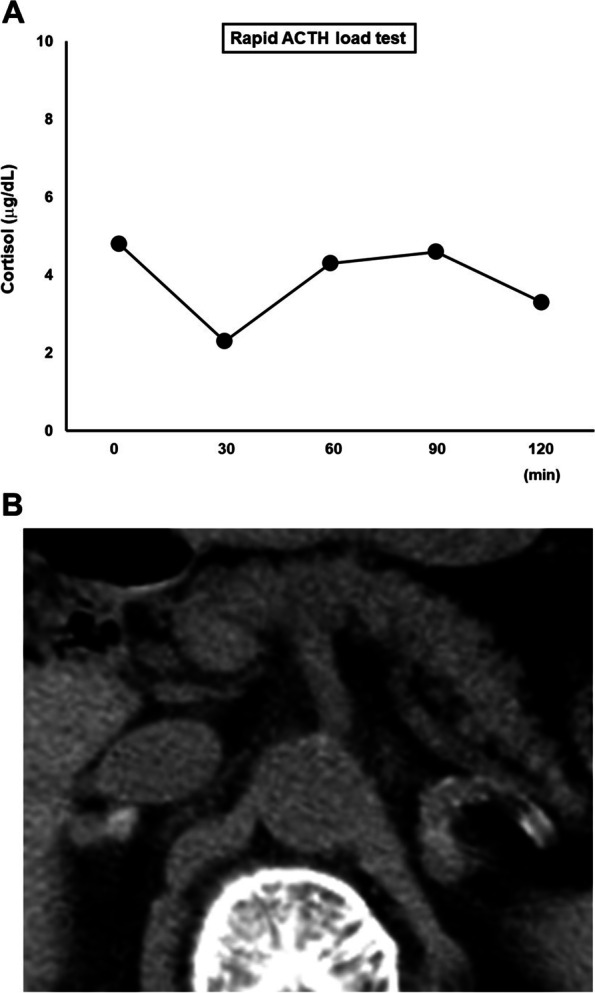
**A** In rapid ACTH load test (0.25 mg of 1–24 ACTH), cortisol level was not increased at all. **B** An abdominal computed tomography (CT) showed swelling of both adrenal glands together with calcification

On admission, her height, body weight and body mass index were 155 cm, 48.8 kg and 20.3 kg/m^2^, respectively. Blood pressure, heart rate and body temperature were 122/72 mmHg, 78 /min and 36.2 °C, respectively. After admission, blood pressure was relatively low (approximately 100–120/50–60 mmHg). Figure 1 shows the picture of her face (Fig. 1A), palm (Fig. 1B), and nail beds in her hands and legs (Fig. 1C, D). There was pigmentation in her face, hands and legs. In particular, dark pigmentation was observed in auricle and nail beds in her hands and legs. Mild pigmentation was observed throughout the whole body. She had type 2 diabetes mellitus and was treated with 0.5 mg of glimepiride and 100 mg of sitagliptin. She had dyslipidemia and was treated with 10 mg of fluvastatin. Table 1 shows the clinical data on admission in this subject. ACTH was high (336.0 pg/mL) (reference range: 7.2–63.3 pg/mL), and cortisol and aldosterone were low as follows: cortisol, 4.8 μg/dL (6.24–18.0 μg/dL); aldosterone < 10 pg/mL (4.0–82.1 pg/mL). Hyponatremia and eosinophilia were observed: sodium, 123 mmol/L (138–145 mmol/L), eosinophils, 12.8% (1.0–5.0%) (white blood cells, 5060 /μL (3300–8600 /μL)). Liver function and renal function were within normal range. Glucose and lipid metabolism were almost normalized with the above-mentioned treatment. Hemoglobin A1c (HbA1c) was 6.2% (4.9–6.0%) and fasting plasma glucose was 95 mg/dL (73–109 mg/dL). In rapid ACTH load test (0.25 mg of 1–24 ACTH), cortisol level was not increased at all (4.8 μg/dL (6.24–18.0 μg/dL)) (Fig. 2A). There was no daily variation in ACTH and cortisol levels. Based on these findings, we diagnosed her with Addison’s disease. After admission, glycemic control was good. It seemed that the presence of adrenal dysfunction increased the possibility of hypoglycemia risk. Therefore, we stopped glimepiride to avoid possible hypoglycemia.

**Table 1:** Clinical data on admission in this subject

Peripheral blood		Reference range	Diabetes markers		Reference range
Red blood cells	396 × 10^4^ /μL	435–555	Plasma glucose	95 mg/dL	73–109
Hemoglobin	12.3 g/dL	13.7–16.8	HbA1c	6.2%	4.9–6.0
White blood cells	5060 /μL	3300–8600			
Neutrophils	39.6%	50.0–70.0	Lipid markers		
Eosinophils	12.8%	1.0–5.0	LDL-cholesterol	93 mg/dL	65–139
Lymphocytes	40.7%	20.0–40.0	HDL-cholesterol	33 mg/dL	40–90
Platelet	17 × 10^4^ /μL	15.8–34.8	Triglyceride	98 mg/dL	40–149
Blood biochemistry			Endocrine markers		
Total protein	6.1 g/dL	6.6–8.1	ACTH	336.0 pg/mL	7.2–63.3
Albumin	3.6 g/dL	4.1–5.1	Cortisol	4.8 μg/dL	6.24–18.0
Total bilirubin	0.7 mg/dL	0.4–1.5	DHEA-S	< 2 μg/dL	76–386
AST	27 U/L	13–30	PRA	8.0 ng/mL/h	0.2–3.9
ALT	14 U/L	10–42	PAC	< 10.0 pg/mL	4.0–82.1
γ-GTP	21 U/L	13–64			
LDH	226 U/L	124–222	Inflammation marker		
Creatinine	0.53 mg/dL	0.65–1.07	CRP	0.31 mg/dL	< 0.14
BUN	8 mg/dL	8–20			
Electrolytes			Urinalysis		
Sodium	123 mEq/L	138–145	pH	7.0	5.0–7.5
Potassium	4.4 mEq/L	3.6–4.8	Protein	(−)	(−)
Chloride	98 mEq/L	101–108	Glucose	(−)	(−)
Calcium	8.3 mg/dL	8.8–10.1	Ketone body	(±)	(−)
Corrected calcium	8.7 mg/dL		Occult blood	(−)	(−)
Phosphorous	2.5 mg/dL	2.7–4.6	Bilirubin	(−)	(−)
Magnesium	2.4 mg/dL	1.9–2.6			

*Abbreviation*: *AST* aspartate aminotransferase, *ALT* alanine aminotransferase, *γ-GTP* γ-glutamyl transpeptidase, *LDH* lactate dehydrogenase, *BUN* blood urea nitrogen, *HbA1c* hemoglobin A1c, *LDL* low density lipoprotein, *HDL* high density lipoprotein, *ACTH* adrenocorticotropic hormone, *DHEA-S* dehydroepiandrosterone sulfate, *PRA* plasma renin activity, *PAC* plasma aldosterone concentration, *CRP* C-reactive protein

**Fig. 3:**
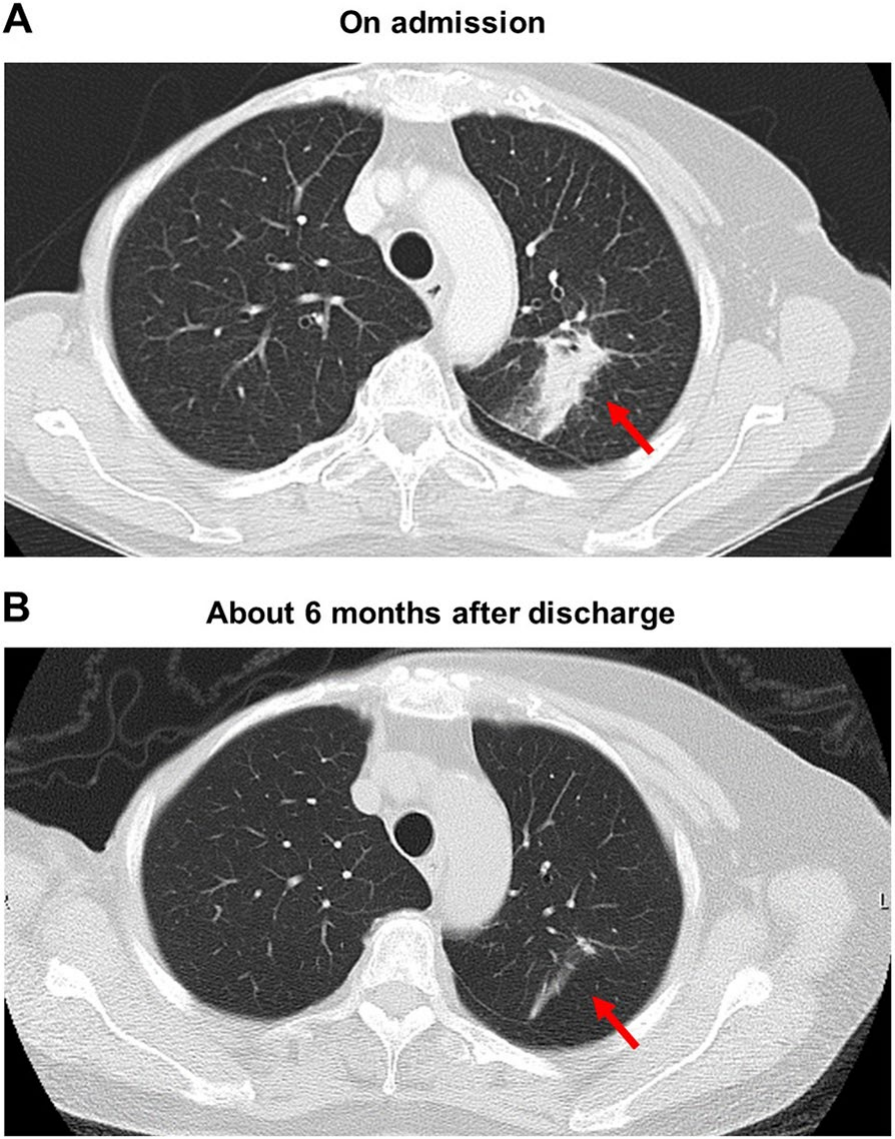
**A** A chest CT on admission showed marked consolidation in the upper lobe of the left lung. **B** A chest CT taken about 6 months later showed drastic reduction of the consolidation in the left lung compared to that on admission

An abdominal computed tomography (CT) showed swelling of both adrenal glands accompanied by calcification (Fig. 2B). We suspected her of having adrenal tuberculosis and performed QuantiFERON test. However, QuantiFERON test was negative and she had no past or family history of tuberculosis. These data ruled out the possibility of adrenal tuberculosis. A chest CT showed marked consolidation in the upper lobe of the left lung (Fig. 3A). Saturation of percutaneous oxygen (SpO_2_) was 97%. We thought that she had non-tuberculous mycobacterial infection and that adrenal failure was brought about by the infection. Since it is known that MAC pulmonary disease accounts for the majority of non-tuberculous mycobacterial infection (more than 90% in Japan), we checked anti-MAC antibody. In this subject, however, anti-MAC antibody was negative. There is idiopathic Addison’s disease which is usually induced by autoimmunity, but in subjects with the disease, calcification is seldom observed in the adrenal gland and adrenal gland often tends to be atrophied. In consideration of these points, it was not likely that this subject had idiopathic Addison’s disease. We started treatment with 10 mg of hydrocortisone and increased it to 20 mg. Sodium level was increased up to 142 mmol/L (138–145 mmol/L), and eosinophil number was decreased from 647 /μL to 309 /μL (33–430 /μL) 4 days after starting hydrocortisone therapy. Appetite loss was markedly reduced at that time, and she was finally discharged from our institution.

Twenty days after discharge, bronchoscopy was performed. In polymerase chain reaction (PCR) test using bronchial lung lavage fluid, *mycobacterium tuberculosis* complex was negative. It is known that MAC pulmonary disease is induced mainly by infection with *mycobacterium avium* or *mycobacterium intracellulare*. In this subject, however, *mycobacterium avium* and mycobacterium intracellular were both negative in PCR test. *Mycobacterium abscessus* was positive in bronchial lung lavage fluid culture. We finally diagnosed this subject with *mycobacterium abscessus* (Mab) pulmonary disease and Addison’s disease triggered by the infection.

A chest CT taken about 6 months later showed drastic reduction of the consolidation in the upper lobe of the left lung (Fig. 3B). Adrenal function was normalized at that time (ACTH, 44.6 pg/mL; cortisol, 8.2 μg/dL). Calcification in the adrenal gland was still observed. Therefore, we continued hydrocortisone therapy (15 mg of hydrocortisone) for about 6 months after discharge.

## Discussion and conclusions

In this case report, we showed a subject who had non-tuberculous mycobacterial infection and Addison’s disease triggered by the infection. It is known that MAC pulmonary disease accounts for a majority of lung non-tuberculous mycobacterial infection. In this subject, however, pathogen bacteria were *mycobacterium abscessus* (Mab), but not MAC. It is noted here that Mab is very rare as a cause of Addison’s disease. Addison’s disease is primary adrenal failure in which cortisol secretion from the adrenal gland is markedly reduced. In this subject, it is likely that the development of calcification was closely associated with destruction of the adrenal gland. Although the consolidation in the left lung was drastically reduced in this subject, endogenous adrenal function was not recovered even after steroid therapy. Therefore, we continued hydrocortisone therapy. It seems that once calcification is developed to some extent, it is difficult to restore adrenal function. We assume that if we had found Addison’s disease at an earlier stage before the development of calcification in the adrenal gland, we might have, at least in part, restored adrenal function to some extent in this subject.

Infection, especially tuberculous infection, is one of main causes of Addison’s disease. In typical Addison’s disease due to adrenal gland tuberculous, swelling of both adrenal glands together with calcification is observed in some imaging examination. Therefore, we suspected this subject of having adrenal tuberculosis, but she had no past or family history of tuberculosis and QuantiFERON test was negative. In PCR test using bronchial lung lavage fluid, *mycobacterium tuberculosis* complex was negative. These findings ruled out the possibility of adrenal tuberculosis. It is known that MAC pulmonary disease accounts for the majority of non-tuberculous mycobacterial infection. Therefore, we suspected her of having MAC pulmonary disease, but anti-MAC antibody was negative. It is also known that *mycobacterium avium* and *mycobacterium intracellulare* are main pathogen bacteria of MAC pulmonary disease. In this subject, however, both bacteria were negative in PCR test using bronchial lung lavage fluid. These findings ruled out the possibility that this subject had MAC pulmonary disease. *Mycobacterium abscessus* (Mab) was positive in bronchial lung lavage fluid culture. We finally diagnosed this subject with Addison’s disease triggered by infection with Mab, but not by tuberculous mycobacterial infection or MAC pulmonary disease. If adrenal gland biopsy had been performed, the biopsy data would have strengthened the above-mentioned diagnosis. However, we did not perform the biopsy in consideration of her age (83 years old), possible risk of infection and the hope of this subject that she did not want us to perform such biopsy.

The incidence of Mab pulmonary disease is slightly increasing, but it is still very rare [6–10]. Indeed, it was reported that Mab infection accounted for only 3% in non-tuberculous mycobacteriosis [5]. Mab infection is more often observed in female compared to male, although its reason remains unknown. Mab infection is usually treated with various kind of antibiotics, and it is often difficult to appropriately control the infection especially in subjects with serious underlying disease who are compromised host [11–13]. In serious cases, surgical operation is needed to treat Mab pulmonary disease. MAC infection is often found as an underlying disease of Mab pulmonary disease. In this subject, after starting hydrocortisone therapy, sodium level was increased and eosinophil number was decreased and her symptoms were markedly mitigated. Finally, she was discharged. *Mycobacterium abscessus* was detected in bronchial lung lavage fluid culture 20 days after discharge. She had no subjective symptoms at that time. Mab infection is usually treated with antibiotics. It has been reported, however, that Mab pulmonary disease is not aggravated even without any treatment in some subjects without serious underlying disease [11–13]. Therefore, we did not start any therapy with antibiotics in this subject. Fludrocortisone is sometimes used in subjects with Addison’s disease, but since salt intake in Japanese is higher compared to other countries, hydrocortisone, but not fludrocortisone, is used as a first-line drug in Japan. Therefore, we used hydrocortisone. However, Mab pulmonary disease in this subject was mitigated without any specific treatment. Since Mab pulmonary disease is sub-classified into three subtypes (*M. abscessus* subsp. abscessus，*M. abscessus* subsp. massiliense，*M. abscessus* subsp. Bolletii) based on gene sequence analysis [14], we assume that the prognosis, at least in part, depends on such subtype of bacteria. It has been also reported that the prognosis depends on host immune response in each subject. For example, when subjects have diabetes mellitus with poor glycemic control, the prognosis of Mab pulmonary disease would be poor. This subject had type 2 diabetes mellitus but glycemic control was good (HbA1c, 6.2%). We assume that good glycemic control was associated with the recovery from Mab pulmonary disease without any specific treatment. As described above, MAC infection is often found as an underlying disease of Mab pulmonary disease, and it is known that bacteria can change from MAC to Mab. In this subject, however, since anti-MAC antibody was negative. We think that this ruled out the possibility that this subject suffered from MAC infection.

There is a limitation in this case report. Autoimmune origin of adrenal insufficiency is the most common cause for adrenal failure in developed countries. In the present case, autoimmunity was excluded based upon CT adrenal image. We failed to examine serum level of adrenal autoantibody. We should have examined adrenal antibody in this subject in order to completely exclude the possibility of autoimmunity.

Taken together, we should bear in mind the possibility of Addison’s disease triggered by non-tuberculous mycobacterial infection. We should check other pathogen bacteria carefully even when we exclude the possibility of adrenal tuberculous and MAC pulmonary disease.

## Data Availability

All datasets on which the conclusions of the paper rely is described in this manuscript and is available to readers.

## Abbreviations

ACTH: adrenocorticotropic hormone
CT: computed tomography
MAC: *mycobacterium avium* complex
PCR: polymerase chain reaction
Mab: *mycobacterium abscessus*
